# Association between Atrial Fibrillation, Myocardial Infarction, Heart Failure and Mortality in Patients with Nontuberculous Mycobacterial Infection: a nationwide population-based study

**DOI:** 10.1038/s41598-019-51801-w

**Published:** 2019-10-29

**Authors:** Chan Soon Park, Eue-Keun Choi, Bongseong Kim, Kyung-Do Han, So-Ryoung Lee, Myung-Jin Cha, Seil Oh

**Affiliations:** 1 Department of Internal Medicine, Seoul National University Hospital, Seoul, Republic of Korea; 2 Graduate School of Medical Science and Engineering, Korea Advanced Institute of Science and Technology, Daejeon, Republic of Korea; 3 Department of Statistics and Actuarial Science, Soongsil University, Seoul, Republic of Korea; 4 Department of Biostatistics, College of Medicine, The Catholic University of Korea, Seoul, Korea

**Keywords:** Interventional cardiology, Risk factors

## Abstract

NTM infection demonstrates an increasing incidence and prevalence. We studied the impact of NTM in cardiovascular events. Using the Korean nationwide database, we included newly diagnosed 1,730 NTM patients between 2005 and 2008 and followed up for new-onset atrial fibrillation (AF), myocardial infarction (MI), heart failure (HF), ischemic stroke (IS), and death. Covariates-matched non-NTM subjects (1:5, n = 8,650) were selected and analyzed. Also, NTM infection was classified into indolent or progressive NTM for risk stratification. During 4.16 ± 1.15 years of the follow-up period, AF, MI, HF, IS, and death were newly diagnosed in 87, 125, 121, 162, and 468 patients. In multivariate analysis, NTM group showed an increased risk of AF (hazard ratio [HR] 2.307, 95% confidence interval [CI] 1.560–3.412) and all-cause death (HR 1.751, 95% CI 1.412–2.172) compared to non-NTM subjects, whereas no significant difference in MI (HR 0.868, 95% CI 0.461–1.634), HF (HR 1.259, 95% CI 0.896–2.016), and IS (HR 1.429, 95% CI 0.981–2.080). After stratification, 1,730 NTM patients were stratified into 1,375 (79.5%) indolent NTM group and 355 (20.5%) progressive NTM group. Progressive NTM showed an increased risk of AF and mortality than indolent NTM group. Screening for AF and IS prevention would be appropriate in these high-risk patients.

## Introduction

Nontuberculous mycobacteria (NTM) are a widespread pathogen in the environment and consist of various species^1^. While NTM can cause lymphadenitis, skin infections, and catheter-related infections, pulmonary infection with NTM is the most common clinical presentation^1–3^. The worldwide incidence of pulmonary infection with NTM has significantly increased^3–6^, and the burden now exceeds that of *Mycobacterium tuberculosis* in some countries^5,7^. Also, NTM-related mortality and incidence rates are increasing^8^.

Cardiovascular (CV) events including myocardial infarction (MI), atrial fibrillation (AF), heart failure (HF), and ischemic stroke (IS) are associated with substantial morbidity and mortality worldwide^9–12^. Efforts have been made to identify and manage potential risk factors to improve clinical outcomes and reduce disease burden. Infection and inflammation are well-known causes of CV disease^13–15^, and various inflammatory diseases have shown an association with CV events^16,17^. In spite of the increasing importance of NTM infection, however, few studies have examined the relationship between NTM infection and CV events.

NTM infection can be either indolent or aggressively progressive^1,18^. Progressive NTM lung disease should be treated with medication while indolent NTM infection can be observed over time^19^. It is unclear whether this difference in severity is associated with the occurrence of CV events.

This study investigated the prognostic significance of NTM infection for CV events and all-cause mortality. We hypothesized that the association between NTM and CV events would be more prominent in patients with progressive NTM infection than in those with indolent NTM infection.

## Results

### Incidence and prevalence of NTM infection

The incidence of NTM infection in Korea consistently increased. Between 2005 and 2015, 24,413 patients were newly diagnosed with NTM infection. The annual incidence of NTM infection gradually increased and peaked in 2015 (20.884 per 100,000 person-years). The incidence was greater in females than in males, and in older groups than in younger counterparts for all study periods. Owing to the gradual increase in NTM incidence, the prevalence of NTM infection also consistently increased. The prevalence of NTM infection increased from 0.906 per 100,000 in 2005 to 26.483 per 100,000 in 2015. When analyzed according to sex and age group, NTM prevalence increased in both sexes, with a female preponderance, and across all age groups.

### Characteristics of the study population

During the enrolment period, 1,730 patients without a previous history of NTM, MI, AF, or HF were newly diagnosed with NTM infection. Among patients with NTM, 1,375 (79.5%) had indolent infection and 355 (20.5%) had progressive infection. The characteristics of the baseline population according to NTM infection are shown in Supplemental Table 1. The mean age in the NTM infection group was 52.8 ± 14.4 years, and 628 (36.3%) were males. Among NTM patients, 7.2% had diabetes mellitus, 25.3% had hypertension, and 13.3% had dyslipidemia; the differences were not significant between those with and without NTM infection. However, chronic obstructive lung disease (30.5% vs. 10.5%, *P* < 0.001) and peripheral artery disease (9.8% vs. 5.7%, *P* < 0.001) were more frequently observed in those with NTM infection than in those without infection.

After 1:5 matching, 8,650 subjects were classified in the non-NTM infection group. As shown in Table 1, all clinical variables were well balanced between the non-NTM and NTM group after matching. Successful matching was validated by estimating standardized differences in baseline characteristics.

**Table 1:** Baseline characteristics of the matched cohort.

	Without NTM infection (n = 8,650)	NTM infection (n = 1,730)	p-value	Absolute standardized difference
**Demographic data**				
Age	52.8 ± 14.4	52.8 ± 14.4	1.000	<0.001
20–39	1,535 (17.8)	307 (17.8)	1.000	<0.001
40–59	4,015 (46.4)	803 (46.4)		
60-	3,100 (35.8)	620 (35.8)		
Male	3,140 (36.3)	628 (36.3)		
**Past medical history**				
Diabetes mellitus	625 (7.2)	125 (7.2)	1.000	<0.001
Hypertension	2,185 (25.3)	437 (25.3)	1.000	<0.001
Dyslipidemia	1,150 (13.3)	230 (13.3)	1.000	<0.001
Chronic obstructive pulmonary disease	2,635 (30.5)	527 (30.5)	1.000	<0.001
Peripheral artery disease	850 (9.8)	170 (9.8)	1.000	<0.001
End stage renal disease	10 (0.1)	2 (0.1)	1.000	<0.001
**Social history**				
Low income level	1,615 (18.7)	323 (18.7)	1.000	<0.001
Smoking history			1.000	<0.001
Never smoking	6,930 (80.1)	1,386 (80.1)		
Ex-smoking	700 (8.1)	140 (8.1)		
Current smoking	1,020 (11.8)	204 (11.8)		
**Follow-up**				
Duration of follow-up (year)	4.1 ± 1.2	3.6 ± 1.2	<0.001	0.407

NTM, nontuberculous mycobacteria.

### Association between NTM infection and CV events

**Figure 1:**
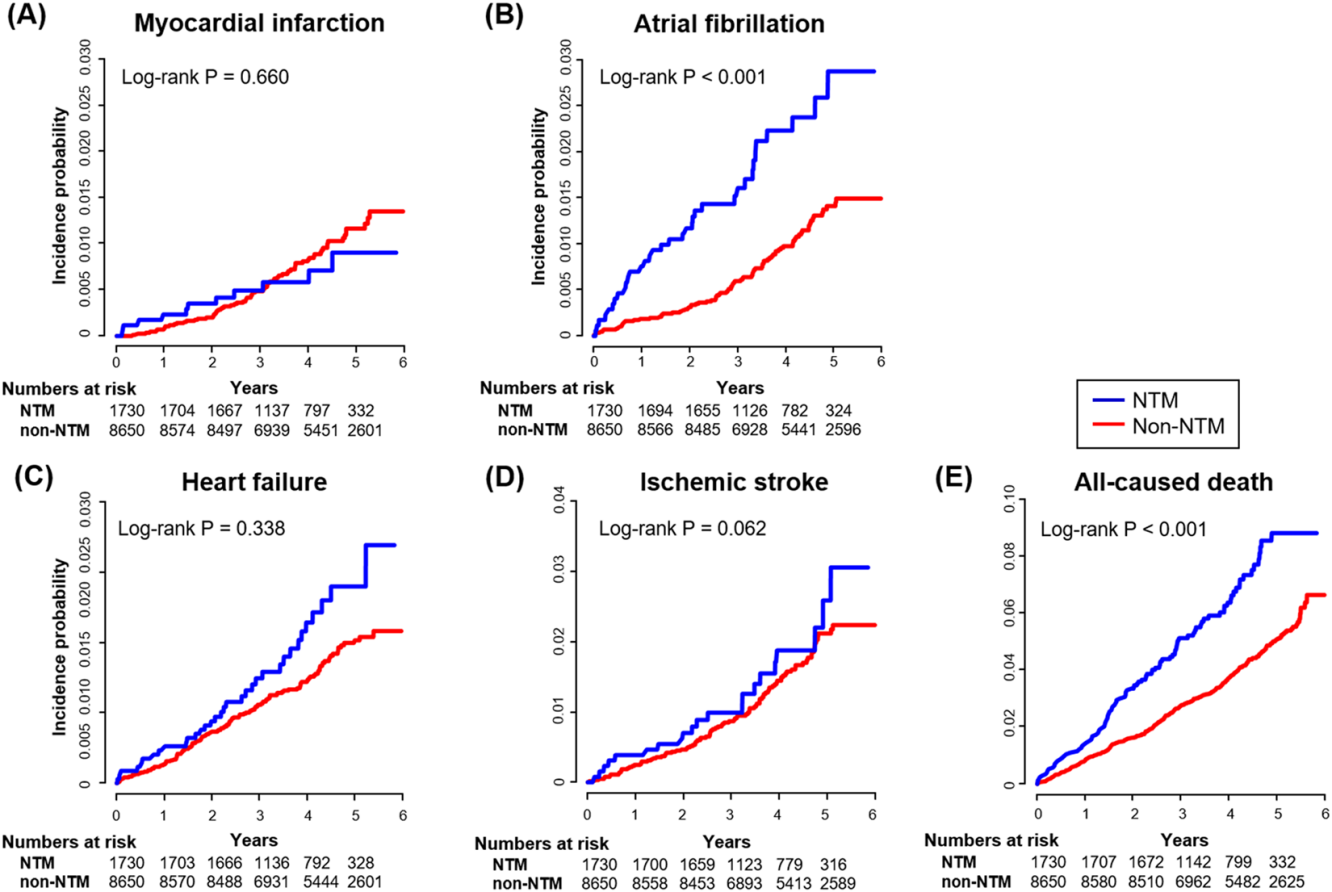
Cardiovascular outcomes according to NTM infection. The Kaplan-Meier survival curves for myocardial infarction, atrial fibrillation, heart failure, ischemic stroke, and all-cause death are presented. Patients with NTM infection showed a higher risk of atrial fibrillation and mortality than those without infection, but not myocardial infarction or heart failure. NTM, nontuberculous mycobacteria.

In Kaplan-Meier analysis, increases in AF and all-cause death (both, log-rank test *P* < 0.001) were frequently observed in patients with NTM. However, there was no significant difference in the incidence of MI (log-rank *P* = 0.660), and HF (log-rank *P* = 0.338) (Fig. 1A–D).

During 4.16 ± 1.15 years of follow-up, 11 cases of MI (IR 1.76 per 1,000 person-years), 35 cases of AF (IR 5.65 per 1,000 person-years), 21 cases of HF (IR 3.36 per 1,000 person-years), 34 cases of IS (IR 5.49 per 1,000 person-years), and 108 cases of death from any cause (IR 17.24 per 1,000 person-years) were observed in the NTM group. In contrast, 76 cases of MI (IR 2.14 per 1,000 person-years), 90 cases of AF (IR 2.54 per 1,000 person-years), 100 cases of HF (IR 2.82 per 1,000 person-years), 138 cases of IS (IR 3.91 per 1,000 person-years), and 360 cases of death from any cause (IR 10.13 per 1,000 person-years) were observed in the non-NTM group. Table 2 shows the number of endpoints, estimated IRs, and HR for CV events. In matched cohorts, the NTM infection group showed an increased risk of AF (HR 2.307, 95% CI 1.560–3.412, *P* < 0.001), and all-cause death (HR 1.751, 95% CI 1.412–2.172), while statistical significance was not observed for differences in MI (HR 0.868, 95% CI 0.461–1.634), HF (HR 1.259, 95% CI 0.896–2.016), and IS (HR 1.429, 95% CI 0.981–2.080). Supplemental Table 2 shows multivariate-adjusted analyses for the baseline population, and the results are consistent with those in Table 2.

**Table 2:** Risk of cardiovascular events according to NTM infection in the matched cohort.

NTM infection	Number	Events	Duration	Incidence rate*	HR (95% CI)	P-value
**Myocardial infarction**						
No	8,650	76	35,453	2.144	1 (reference)	0.661
Yes	1,730	11	6,250	1.760	0.868 (0.461–1.634)	
**Atrial fibrillation**						
No	8,650	90	35,415	1.609	1 (reference)	<0.001
Yes	1,730	35	6,197	5.648	2.307 (1.560–3.412)	
**Heart failure**						
No	8,650	100	35,429	1.582	1 (reference)	0.339
Yes	1,730	21	6,240	3.365	1.259 (0.896–2.016)	
**Ischemic stroke**						
No	8,650	138	35,290	3.910	1 (reference)	0.063
Yes	1,730	24	6,195	5.488	1.429 (0.981–2.080)	
**Death**						
No	8,650	360	35,550	4.968	1 (reference)	<0.001
Yes	1,730	108	6,266	17.235	1.751 (1.412–2.172)	

CI, confidence interval; HR, hazard ratio; NTM, nontuberculous mycobacteria.

*Incidence rates were calculated per 1,000 patient-years from matched cohort.

### Risk of CV events according to the severity of NTM infection

We also investigated the risk of CV events according to infection severity stratification. The demographic features of non-NTM, indolent NTM, and progressive NTM groups are shown in Supplemental Table 3. Patients in the progressive NTM group were older and more likely to have a previous history of chronic obstructive pulmonary artery disease and peripheral artery disease. The prevalence of diabetes mellitus, hypertension, and dyslipidemia were not different across groups.

**Figure 2:**
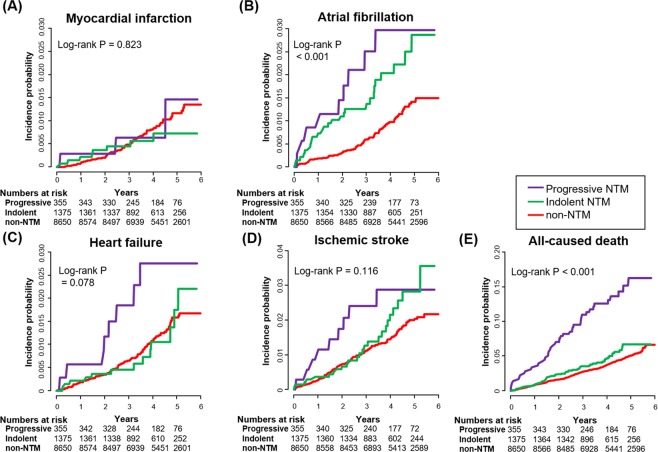
Cardiovascular outcomes according to indolent and progressive NTM infection. Kaplan-Meier survival analyses were performed to evaluate the prognostic impact on myocardial infarction, atrial fibrillation, heart failure, ischemic stroke, and all-cause death according to indolent and progressive NTM infection. NTM, nontuberculous mycobacteria.

In Kaplan-Meier analysis (Fig. 2A–E), indolent and progressive NTM both showed an increased risk of AF (log-rank *P* < 0.001, respectively) and death (log-rank *P* < 0.001, respectively). Interestingly, the progressive NTM group showed a higher risk of mortality than the indolent NTM group (log-rank *P* = 0.003). The progressive NTM group showed a nonsignificant trend toward increased risk of HF and IS compared to that in the indolent NTM or control (log-rank *P* = 0.078 and log-rank *P* = 0.116) groups. However, there was no association between indolent or progressive NTM and the risk of MI. These results were also observed when analyzed with Cox regression models, as shown in Supplemental Table 4. After adjusting for covariates in the baseline population, the indolent and progressive NTM groups demonstrated significant associations with AF (HR 2.207, 95% CI 1.502–3.241 and HR 2.217, 95% CI 1.154–4.261, respectively) and all-cause death (HR 3.248, 95% CI 2.433–4.336 and HR 1.486, 95% CI 1.159–1.906), but not with MI or HF.

### Subgroup analysis

We performed a subgroup analysis based on CV risk factors: age, sex, diabetes mellitus, hypertension, dyslipidemia, chronic obstructive pulmonary disease, income level, and smoking history. Analysis of those with NTM infection demonstrated higher risks of AF and mortality in most of the exploratory subgroups and similar risks of MI, HF, and IS (Supplemental Fig. A–E). There was no observed significant interaction between subgroups of patients.

## Discussion

The major findings of our study were as follows: (1) the annual incidence and prevalence of NTM infection gradually increased between 2005 and 2015; (2) those with NTM infection showed an increased risk of AF and death but not MI, HF, and IS, compared to those without NTM infection; (3) about 20% of NTM patients were classified as having progressive NTM, which requires antibiotic treatment; and (4) the progressive NTM group showed an increased risk of AF and death compared to that in the indolent NTM group, but there was no significant difference in the risk of MI, HF, and IS between the groups.

Although we did not explore the mechanism of the relationship between infection and CV events, there are several possible explanations for the increased risk in patients with NTM. Previous studies reported that CV events were significantly associated with various infectious or inflammatory disease such as pneumonia and psoriasis^13–17,20–22^. Inflammatory cytokines such as C-reactive protein, interleukin-6, and tumor necrosis factor-alpha (TNF-α) showed significant associations with AF^23^. Cytokines and proinflammatory factors also have an impact on the pathophysiology of HF^24^. NTM infection causes chronic pulmonary inflammation, which is difficult to eradicate and requires long-term and sometimes life-long treatment^1,25^. NTM infection may induce changes in inflammatory cells and cytokines that sustain prolonged inflammation^26,27^ and may be associated with an increased risk of CV events.

Additionally, some reports indicated that patients with NTM infection had combined risk factors such as old age, smoking, and chronic obstructive lung disease^1,4^. Given that these are conventional CV risk factors that have consistently shown prognostic value, we could postulate that NTM patients might have a higher risk of CV events. Interestingly, NTM infection showed a significant association with AF, while there was no association between NTM infection and MI, and only a marginal association between NTM infection, HF, and IS. Prognostic differences among CV diseases should be investigated in future studies.

In this study, we not only investigated the association between NTM and CV events but also stratified NTM infection according to severity. As NTM is characterized by a variety of manifestations, we postulated prognostic differences according to the severity of NTM infection and found that progressive NTM infection had a stronger association with CV events than indolent NTM infection.

The prevalence and incidence of NTM infection have surged in both Western and Asian populations^3–6,28^. As urbanization and piped water have been suggested as causes for this global epidemiologic trend, it is conceivable that NTM infection will become an important theme in public health^25,29,30^. Because of the high social burden of CV events in spite of recent advances in treatment^9^, there is a need to identify high-risk subjects and to perform tailored management. However, there is a paucity of data on prognostic associations between NTM infection and CV events. To the best of our knowledge, this study is the first to report that NTM patients – especially those with progressive NTM – had an increased risk of CV events.

Our study had several strengths. First, we performed this analysis using a large, nationwide cohort. Therefore, we had access to many NTM patient records and a follow-up duration adequate for investigation of discrepancies in CV prognosis. Second, because of the variety of clinical manifestations, we further analyzed the association between NTM infection and CV events after stratification according to NTM severity.

Our study had several limitations. First, this was an observational study, and there may have been unmeasured confounders that we did not adjust for. Second, as we used diagnostic codes from claims data, our results might be skewed due to misclassification or underestimation bias. However, the insurance database has been well managed under national supervision. Also, we performed a validation test, which showed a 98.6% positive predictive value. Third, sophisticated research is needed to explain the mechanism of the association between NTM infection and the increased risk of CV events. Fourth, there might be a bias that patients with NTM might visit medical institutes more frequently which could cause increased diagnosis of AF. However, patients with AF usually have significant symptoms, and those symptomatic patients would be finally diagnosed with AF irrespective of routine check-up for other complaints. In addition, 12-lead ECG is not routinely recommended in patients with NTM infection. At last, the risks of CV and non-CV deaths were not presented because the cause of death was not available in our database. In accordance with previous studies based on real-world databases, we only report the results of all-cause deaths as one of the end points^31,32^.

## Conclusions

Using the Korean nationwide health insurance database for this cohort study, we found that NTM infection was associated with AF and mortality. We also found that progressive NTM infection was strongly associated with increased CV risks. These findings suggest the potential benefit of CV surveillance in NTM patients.

## Methods

### Data source and database contents

The Korean National Health Insurance System (NHIS) covers health care services for the entire Korean population of approximately 50 million. The NHIS sponsors a program of regular check-ups for the public at least biennially. Details on the NHIS system have been reported elsewhere^33^. The NHIS maintains claims, health check-up, qualification, and death information databases. This study analyzed information from the claims, health check-up, and death information databases, and was conducted according to the Declaration of Helsinki and approved by the Seoul National University Hospital Institutional Review Board (IRB No. 1801-005-911). The institutional review board waived the need for a written informed consent.

### Establishment of the study cohort

**Figure 3:**
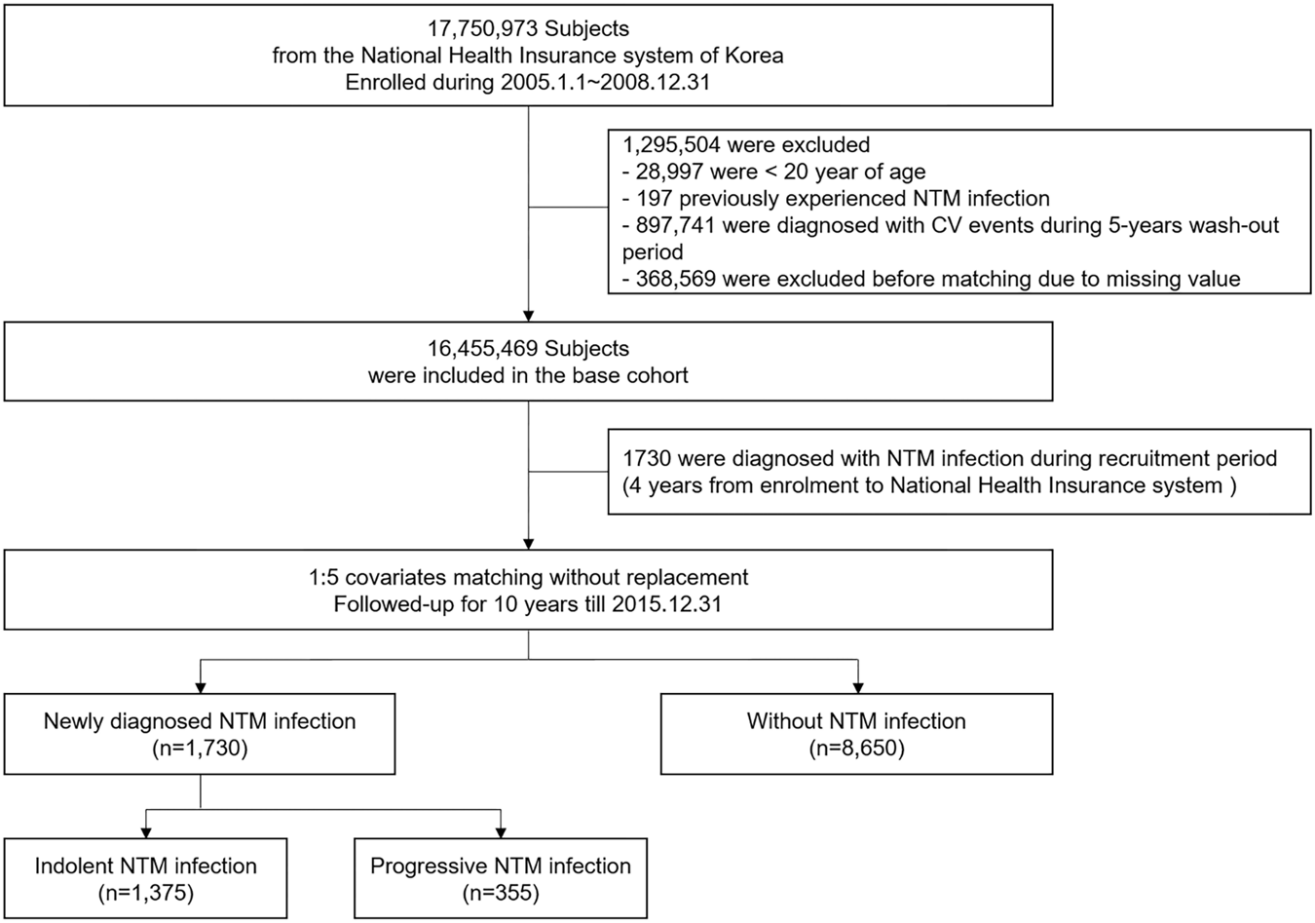
Study flow chart. NTM, nontuberculous mycobacteria.

We initially recruited 17,750,973 Koreans who underwent a baseline health check-up through the NHIS between 2005 and 2008 (Fig. 3). Of these, 17,721,976 were older than 20 years. From this population, we excluded 197 who were diagnosed with NTM infection before the initial health check-up. We then excluded those with a history of more than 1 episode of MI, AF, HF, or IS within the first 5 years after the initial health check-up after the inception of the database, leaving 16,455,469 subjects in the baseline cohort. This policy guaranteed an adequate washout period for previously diagnosed clinical endpoints. Finally, 1,730 patients newly diagnosed with NTM infection were identified during the 4 years of the enrolment period.

Using the baseline cohort, we performed 1:5 matching to adjust for possible confounders, as discrepancies in baseline characteristics could contribute to CV events and biased results. Variables that could affect clinical outcomes included: age, sex, previous history of diabetes mellitus, hypertension, dyslipidemia, chronic obstructive pulmonary disease, peripheral artery disease, end-stage renal disease, income, and smoking history. Matching was conducted for a comparison between patients with and without NTM infection.

### Definition of NTM infection, CV events, and other variables

NTM infection was defined using the ICD-10 codes (A318) registered in the NHIS database by the physician responsible for the treatment. To evaluate the accuracy of our definition of NTM infection, we performed a validation study in our institution for 295 adult patients with ICD-10 code A318. By reviewing the comprehensive medical record, the positive predictive value was found to be 98.6%. Patients with progressive NTM infection were defined as those with both NTM diagnosis and anti-NTM antibiotic treatment, and other patients were defined as those with indolent NTM infection (Supplemental Table 5).

Clinical endpoints were defined as the occurrence of CV events including MI, AF, HF, IS, or death during follow-up. The definition of each CV event was described in our previous reports, and Supplemental Table 5 ^17,34^. Comorbidities such as hypertension, diabetes mellitus, dyslipidemia, chronic obstructive lung disease, peripheral artery disease, and end-stage renal disease were also defined using ICD-10 codes with additional information (Supplemental Table 5). Smoking history was obtained by questionnaire.

### Statistical analysis

Data are presented as numbers and relative frequencies for categorical variables and as a mean ± standard deviation for continuous variables. For comparisons between groups, the chi-square test (or Fisher’s exact test when any expected cell count was <5 for a 2 × 2 table) was adopted for categorical variables, and an unpaired Student’s t-test was performed for continuous variables. The temporal trend of the outcomes was studied by using Kaplan-Meier estimates and compared according to NTM infection. Cox proportional hazard models were used to estimate the hazard ratio (HR) and corresponding 95% confidence interval (CI) for the association between CV endpoints and NTM infection. Each CV event was followed and performed analysis independently except death.

Two-sided *P*-values < 0.05 were considered statistically significant. Statistical tests were performed using SAS version 9.3 (SAS Institute, Cary, NC, USA) and R programming version 3.5.1 (The R Foundation for Statistical Computing, Vienna, Austria, http://www.R-project.org).

## Supplementary information

Supplemental material
