# Supplementary material for: Association between Atrial Fibrillation, Myocardial Infarction, Heart Failure and Mortality in Patients with Nontuberculous Mycobacterial Infection: a nationwide population-based study
Source: Sci Rep. 2019 Oct 29;9:15503. doi: 10.1038/s41598-019-51801-w (PMC6820717; doi:10.1038/s41598-019-51801-w)
Supplement: Supplementary file 1 — Supplemental material [file 41598_2019_51801_MOESM1_ESM.docx]

**Association between Atrial Fibrillation, Myocardial Infarction, Heart Failure and Mortality in Patients with Nontuberculous Mycobacterial Infection: a nationwide population-based study**

Chan Soon Park,^1,2^ Eue-Keun Choi,^1*^ Bongseong Kim,^3^ Kyung-Do Han,^4^ So-Ryoung Lee,^1^ Myung-Jin Cha,^1^ Seil Oh^1^

^1^Department of Internal Medicine, Seoul National University Hospital, Seoul, Republic of Korea

^2^Graduate School of Medical Science and Engineering, Korea Advanced Institute of Science and Technology, Daejeon, Republic of Korea

^3^Department of Statistics and Actuarial Science, Soongsil University, Seoul, Republic of Korea

^4^Department of Biostatistics, College of Medicine, The Catholic University of Korea, Seoul, Korea

**Corresponding Author:**

Eue-Keun Choi, MD, PhD

Professor, Department of Internal Medicine, Seoul National University Hospital,

101 Daehak-ro, Jongno-gu, Seoul, 03080, Republic of Korea

Tel: 82-2-2072-0688

Fax: 82-2-762-9662

E-mail: [choiek17@snu.ac.kr](mailto:choiek17@snu.ac.kr)

**Supplementary material**

**Supplemental figure. Subgroup analyses for cardiovascular risk in NTM patients**

**
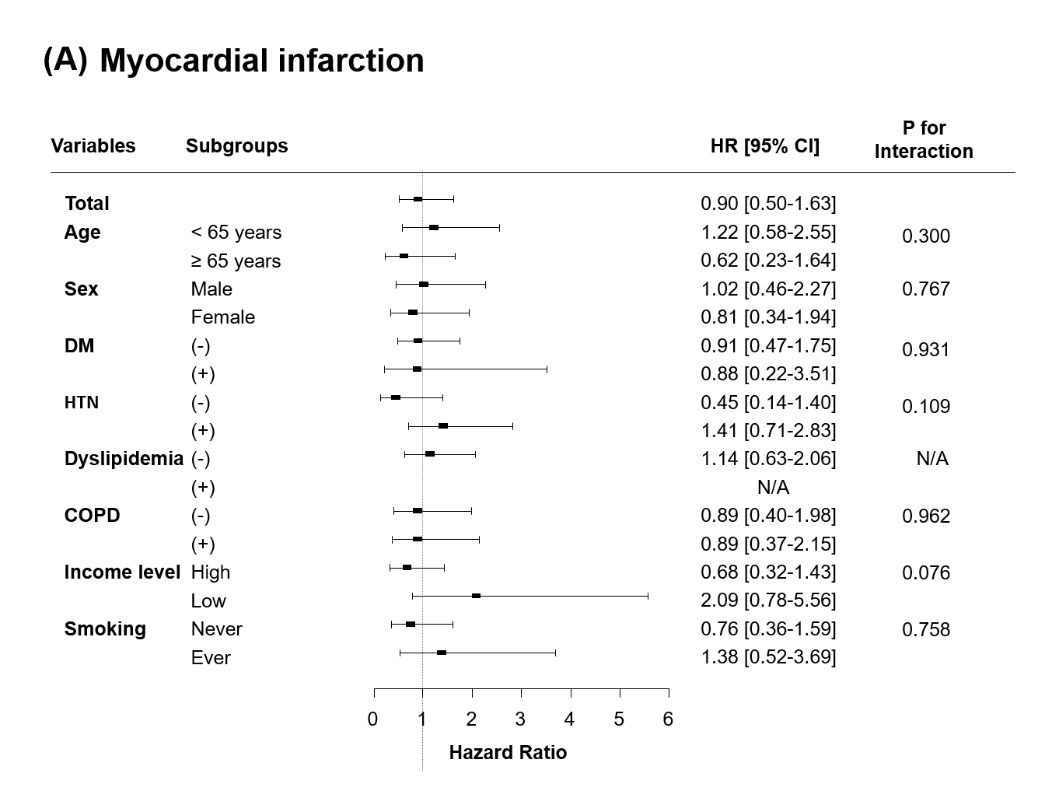
**

**
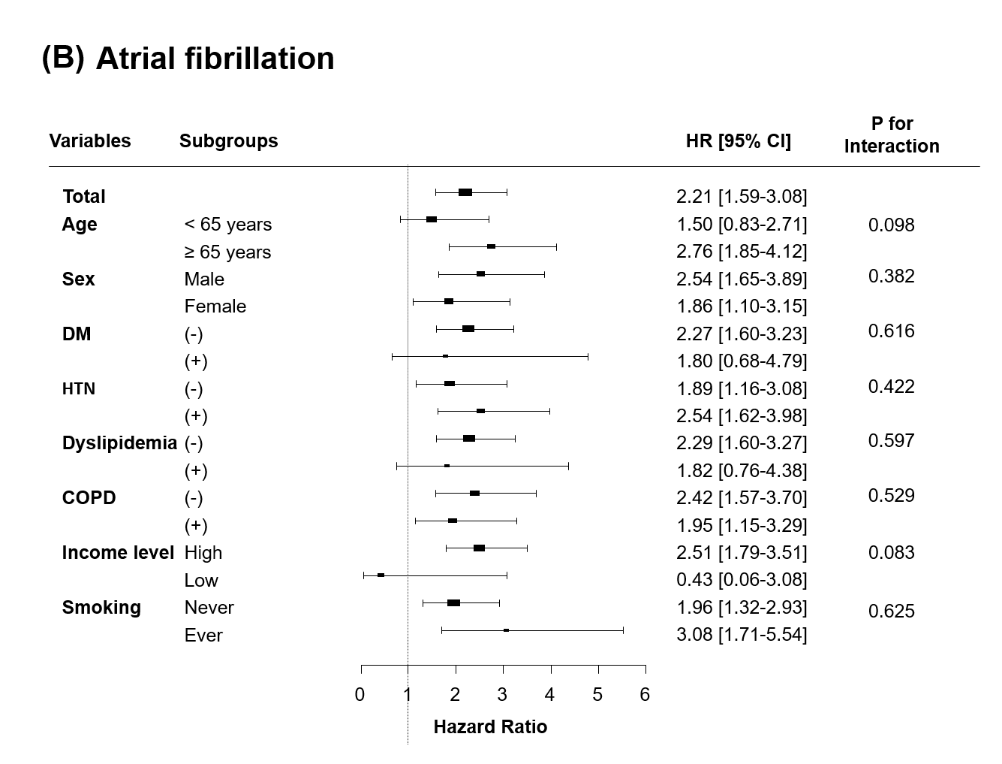
**

**
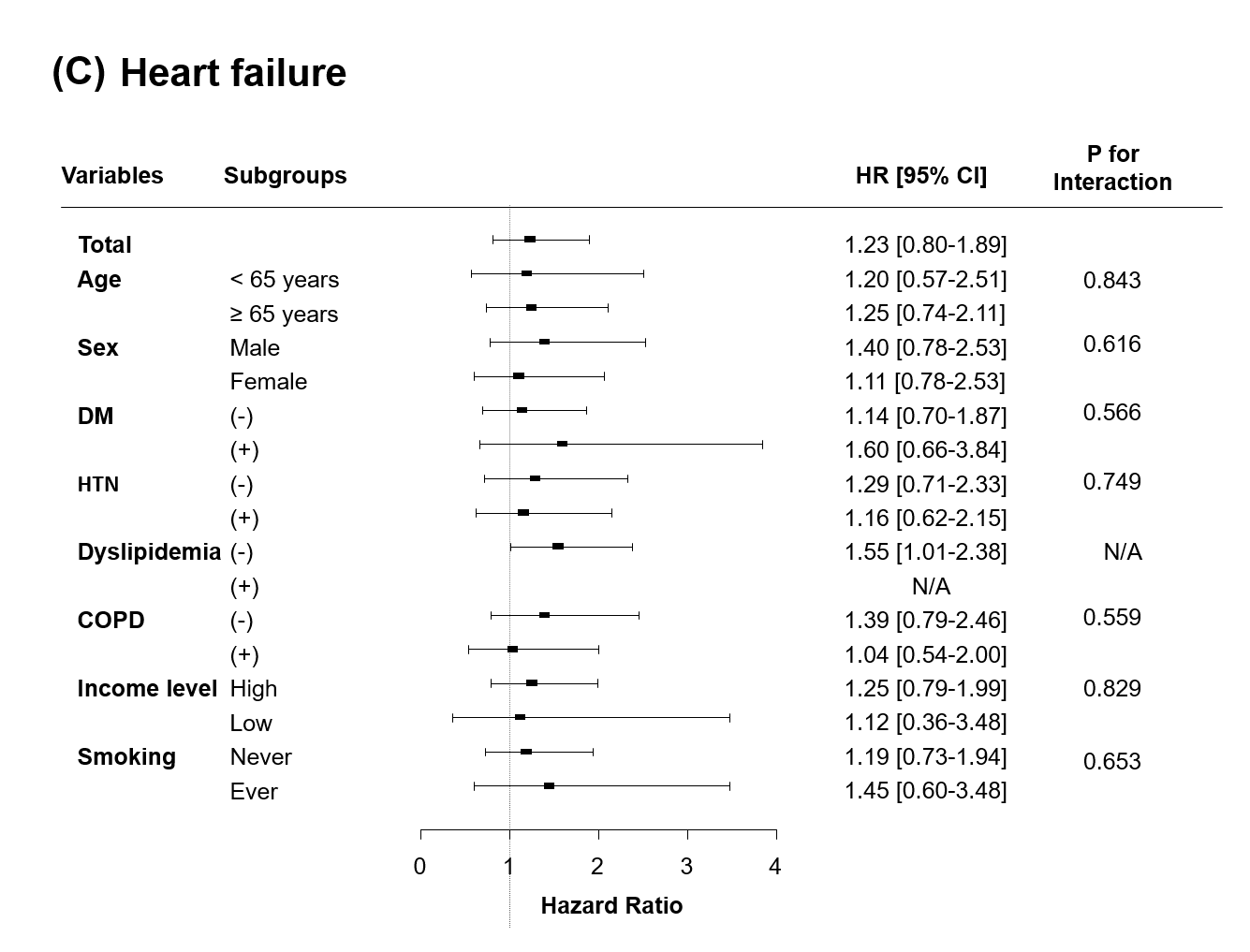

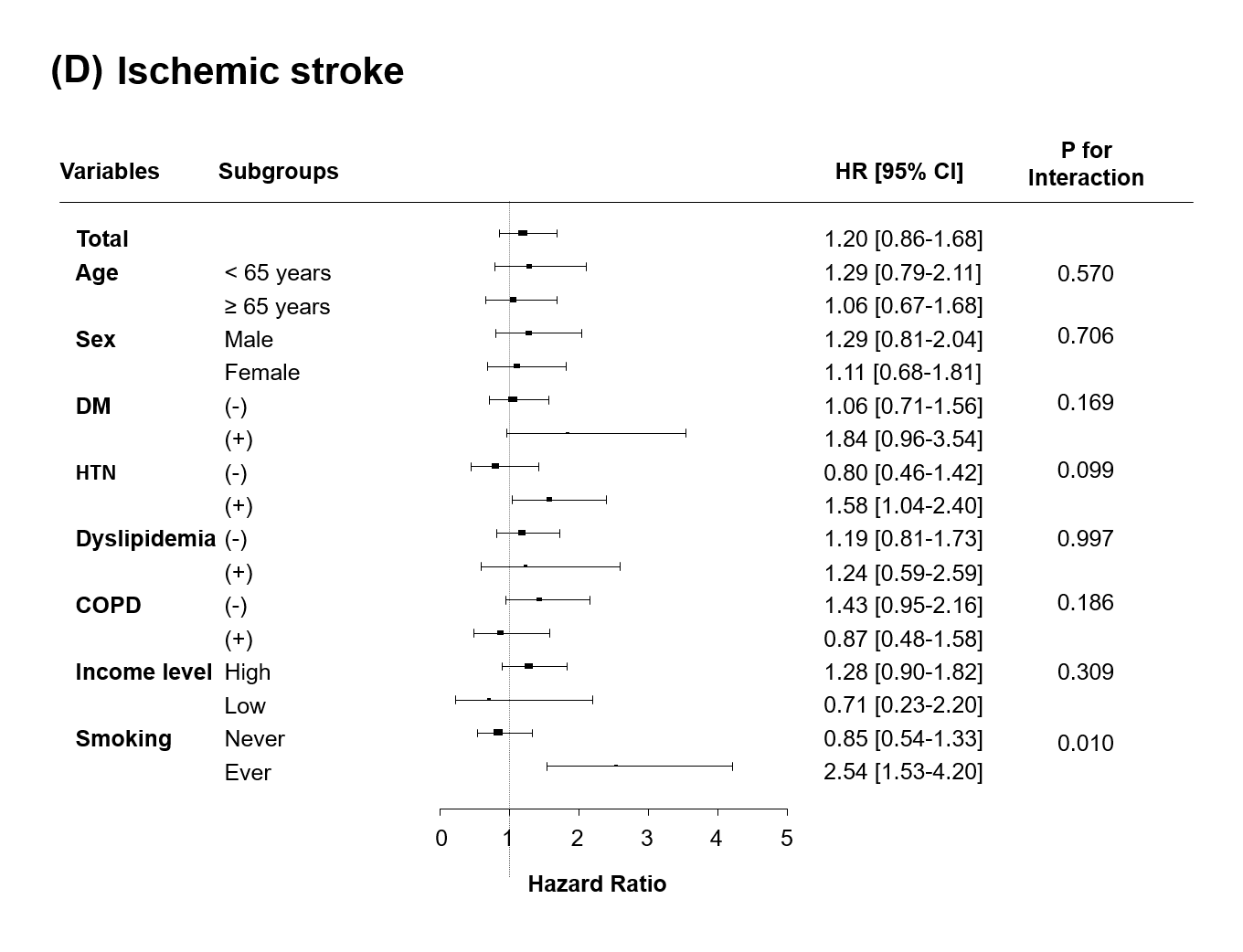

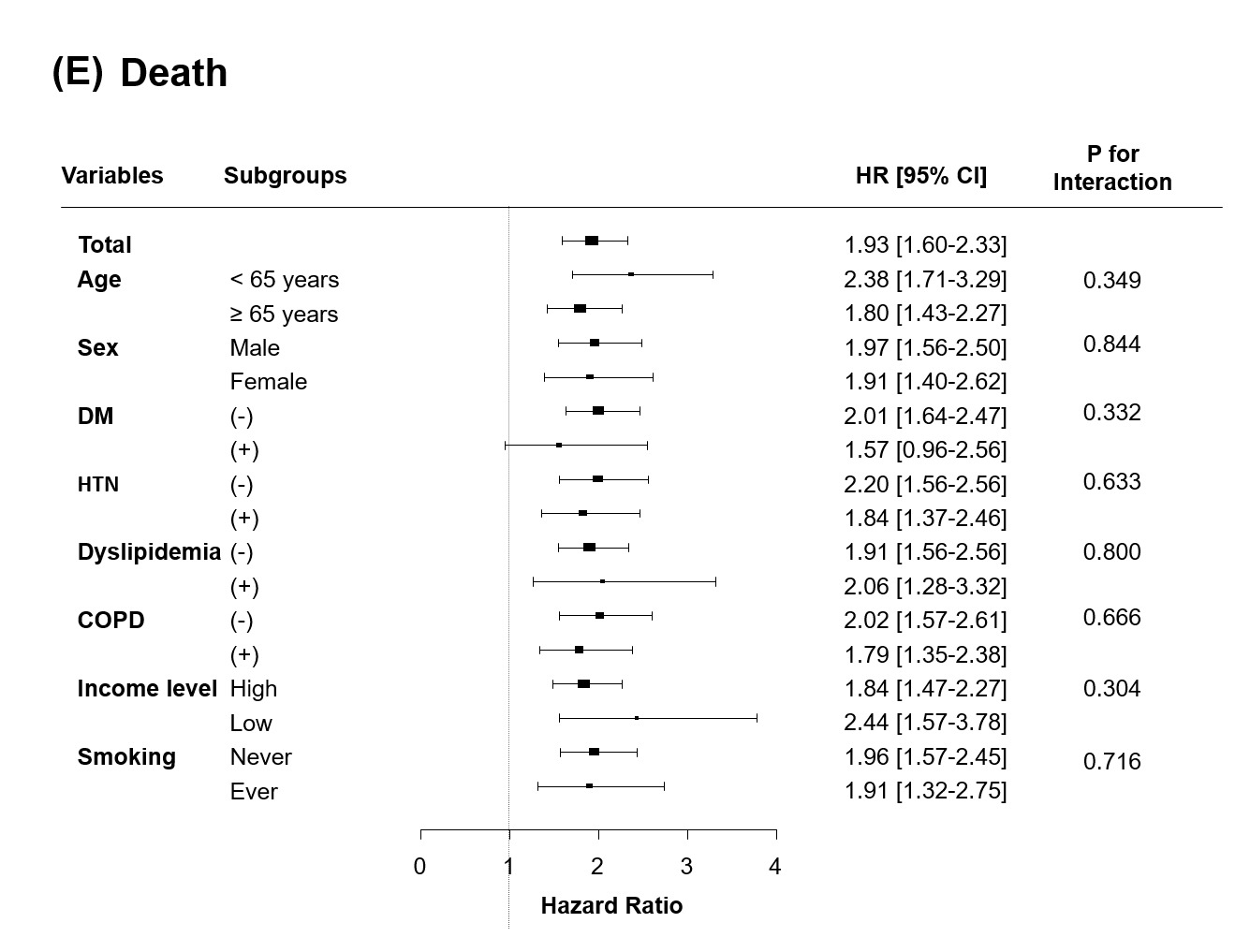
**

CI, confidence interval; COPD, chronic obstructive pulmonary disease; DM, diabetes mellitus; HR, hazard ratio; HTN, hypertension

**Supplemental table 1. Baseline characteristics of the population according to NTM infection**

|  | **Without NTM infection**  **(n=16,453,739)** | **NTM infection**  **(n=1,730)** | **p-value** |  |
| --- | --- | --- | --- | --- |
| **Demographic data** |  |  |  |  |
| Age | 45.1±14.1 | 52.8±14.4 | <0.001 |  |
| 20-39 | 6,101,140 (37.1) | 307 (178.8) |  |  |
| 40-59 | 7,442,442 (45.2) | 803 (46.4) |  |  |
| 60- | 2,910,157 (17.7) | 620 (35.8) |  |  |
| Male | 8,699,690 (52.9) | 628 (36.3) | <0.001 |  |
| **Past medical history** |  |  |  |  |
| Diabetes mellitus | 1,176,172 (7.2) | 125 (7.2) | 0.901 |  |
| Hypertension | 4,067,451 (24.7) | 437 (25.3) | 0.603 |  |
| Dyslipidemia | 2,279,314 (13.9) | 230 (13.3) | 0.502 |  |
| Chronic obstructive pulmonary disease | 1,728,089 (10.5) | 527 (30.5) | <0.001 |  |
| Peripheral artery disease | 934,989 (5.7) | 170 (9.8) | <0.001 |  |
| End stage renal disease | 5,846 (0.04) | 2 (0.1) | 0.078 |  |
| **Social history** |  |  |  |  |
| Low income level | 3,529,434 (21.5) | 323 (18.7) | 0.005 |  |
| Smoking history |  |  | <0.001 |  |
| Never smoking | 11,006,723 (66.9) | 1,386 (80.1) |  |  |
| Ex-smoking | 1,358,973 (8.3) | 140 (8.1) |  |  |
| Current smoking | 4,088,043 (24.9) | 204 (11.8) |  |  |
| **Follow-up** |  |  |  |  |
| Duration of follow-up (year) | 4.2±1.2 | 3.6±1.2 | <0.001 |  |

NTM, nontuberculous mycobacteria

**Supplemental Table 2. Risk of cardiovascular events according to NTM infection in the baseline population**

| **NTM infection** | **Number** | **Events** | **Duration** | **Incidence rate*** | **Unadjusted** | **Multivariate adjusted**^†^ |
| --- | --- | --- | --- | --- | --- | --- |
|  |  |  |  |  | **HR** **(95% CI)** | **HR** **(95% CI)** |
| **Myocardial infarction** | | | | | | |
| No | 16,453,739 | 88,758 | 68,310,394 | 1.299 | 1 (reference) | 1 (reference) |
| Yes | 1,730 | 11 | 6,250 | 1.760 | 1.422 (0.788-2.566) | 0.902 (0.500-1.629) |
| **Atrial fibrillation** | | | | | | |
| No | 16,453,739 | 109,874 | 68,280,611 | 1.609 | 1 (reference) | 1 (reference) |
| Yes | 1,730 | 35 | 6,197 | 5.648 | 3.735 (2.686-5.192) | 2.209 (1.586-3.077) |
| **Heart failure** | | | | | | |
| No | 16,453,739 | 108,078 | 68,309,534 | 1.582 | 1 (reference) | 1 (reference) |
| Yes | 1,730 | 21 | 6,240 | 3.365 | 2.267 (1.479-3.474) | 1.234 (0.805-1.893) |
| **Ischemic stroke** | |  |  |  |  |  |
| No | 16,453,739 | 189,189 | 68,038,979 | 2.781 | 1 (reference) | 1 (reference) |
| Yes | 1730 | 34 | 6,195 | 5.488 | 1.968 (1.406-2.754) | 1.198 (0.856-1.676) |
| **All-cause death** | | | | | | |
| No | 16,453,739 | 340,012 | 68,445,235 | 4.968 | 1 (reference) | 1 (reference) |
| Yes | 1,730 | 108 | 6,266 | 17.235 | 3.556 (2.947-4.291) | 1.932 (1.600-2.333) |

CI, confidence interval; HR, hazard ratio; NTM, nontuberculous mycobacteria

* Incidence rates were calculated per 1000 patient-years from baseline population

^†^ Multivariate-adjusted hazard ratios were calculated by Cox regression models, including age, sex, previous history of diabetes mellitus, hypertension, dyslipidemia, chronic obstructive pulmonary disease, peripheral artery disease, end-stage renal disease, low-income level, and smoking history

**Supplemental Table 3. Baseline characteristics of study subjects according to NTM severity**

|  | **Without NTM infection**  **(n=16,453,739)** | **Indolent NTM infection**  **(n=1,375)** | **Progressive NTM infection**  **(n=355)** | **p-value** |
| --- | --- | --- | --- | --- |
| **Demographic data** |  |  |  |  |
| Age | 45.1±14.1 | 51.4±11.8 | 58.1±11.8 | <0.001 |
| 20-39 | 6,101,140 (37.1) | 286 (20.8) | 21 (5.9) | <0.001 |
| 40-59 | 7,442,442 (45.2) | 644 (46.8) | 159 (44.8) |  |
| 60- | 2,910,157 (17.7) | 445 (32.4) | 175 (49.3) |  |
| Male | 8,699,690 (52.9) | 487 (35.4) | 141 (39.7) | <0.001 |
| **Past medical history** |  |  |  |  |
| Diabetes mellitus | 1,176,172 (7.2) | 101 (7.4) | 24 (6.8) | 0.923 |
| Hypertension | 40,676,451 (24.7) | 336 (24.4) | 101 (28.5) | 0.257 |
| Dyslipidemia | 2,279,314 (13.9) | 183 (13.3) | 47 (13.2) | 0.798 |
| Chronic obstructive pulmonary disease | 1,728,089 (10.5) | 354 (25.8) | 173 (48.7) | <0.001 |
| Peripheral artery disease | 934,989 (5.7) | 125 (9.1) | 45 (12.7) | <0.001 |
| End stage renal disease | 5,846 (0.04) | 2 (0.2) | 0 (0) | 0.091 |
| **Social history** |  |  |  |  |
| Low income level | 3,529,434 (21.5) | 268 (19.5) | 55 (15.5) | 0.005 |
| Smoking history |  |  |  | <0.001 |
| Never smoking | 11,006,723 (66.9) | 1,089 (79.2) | 297 (83.7) |  |
| Ex-smoking | 1,358,973 (8.3) | 111 (8.1) | 29 (8.17) |  |
| Current smoking | 4,088,043 (24.9) | 175 (12.7) | 29 (8.17) |  |
| **Follow-up** |  |  |  |  |
| Duration of follow-up (year) | 4.2±1.2 | 3.6±1.2 | 3.7±1.3 | <0.001 |

NTM, nontuberculous mycobacteria

**Supplemental Table 4. Risk of cardiovascular events according to NTM severity in the baseline population**

| **NTM infection** | **Number** | **Events** | **Duration** | **Incidence rate*** | **Unadjusted** | **Multivariate adjusted**^†^ |
| --- | --- | --- | --- | --- | --- | --- |
|  |  |  |  |  | **HR (95% CI)** | **HR** **(95% CI)** |
| **Myocardial infarction** | | | | | | |
| No | 16,453,739 | 88,758 | 68,310,394 | 1.299 | 1 (reference) | 1 (reference) |
| Indolent | 1,375 | 8 | 4,937 | 5.299 | 1.313 (0.657-2.624) | 0.874 (0.437-1.748) |
| Progressive | 355 | 3 | 1,313 | 6.978 | 1.855 (0.606-5.681) | 0.987 (0.318-3.062) |
| **Atrial fibrillation** | | | | | | |
| No | 16,453,739 | 109,874 | 68,280,611 | 1.609 | 1 (reference) | 1 (reference) |
| Indolent | 1,375 | 26 | 4,907 | 5.299 | 3.480 (2.369-5.111) | 2.207 (1.502-3.241) |
| Progressive | 355 | 9 | 1,290 | 6.978 | 4.504 (2.345-8.653) | 2.217 (1.154-4.261) |
| **Heart failure** | | | | | | |
| No | 16,453,739 | 108,078 | 68,309,534 | 1.582 | 1 (reference) | 1 (reference) |
| Indolent | 1,375 | 13 | 4,933 | 2.635 | 1.777 (1.032-3.060) | 1.032 (0.599-1.777) |
| Progressive | 355 | 8 | 1,308 | 6.118 | 4.136 (2.085-8.202) | 1.812 (0.906-3.624) |
| **Ischemic stroke** | |  |  |  |  |  |
| No | 16,453,739 | 189,189 | 68,038,979 | 2.781 | 1 (ference) | 1 (reference) |
| Indolent | 1,375 | 25 | 1291 | 6.974 | 1.827 (1.234-2.703) | 1.176 (0.794-1.740) |
| Progressive | 355 | 9 | 4905 | 5.097 | 2.525 (1.318-4.838) | 1.262 (0.657-2.426) |
| **All-cause death** | | | | | | |
| No | 16,453,739 | 340,012 | 68,445,235 | 4.968 | 1 (reference) | 1 (reference) |
| Indolent | 1,375 | 62 | 4,952 | 12.520 | 2.575 (2.008-3.302) | 1.486 (1.159-1.906) |
| Progressive | 355 | 46 | 1,314 | 34.998 | 7.146 (5.354-9.539) | 3.248 (2.433-4.336) |

CI, confidence interval; HR, hazard ratio; NTM, nontuberculous mycobacteria

* Incidence rates were calculated per 1000 patient-years from baseline population

^†^ Multivariate-adjusted hazard ratios were calculated by Cox regression models, including age, sex, previous history of diabetes mellitus, hypertension, dyslipidemia, chronic obstructive pulmonary disease, peripheral artery disease, end-stage renal disease, low-income level, and smoking history

**Supplemental table 5. List of the definitions of outcomes and comorbidities**

| **Diagnosis** | **ICD-10-CM code and definition** |
| --- | --- |
| **NTM infection** |  |
| Progressive infection | A318; with minimum one of criteria below fulfilled  -clarithromycin or azithromycin treatment more than 1 month  -isoniazid or rifampicin/rifabutin treatment more than 1 month without a diagnosis of mycobacterial tuberculosis (A15-A19) |
| Indolent infection | A318; not defined as progressive infection |
| **Past medical history** |  |
| Hypertension^a^ | I10-I13, I15; and minimum 1 prescription of anti-hypertensive drug (thiazide, loop diuretics, aldosterone antagonist, alpha-/beta-blocker, calcium-channel blocker, angiotensin-converting enzyme inhibitor, angiotensin II receptor blocker). |
| Diabetes mellitus^a^ | E11-E14; and minimum 1 prescription of anti-diabetic drugs (sulfonylureas, metformin, meglitinides, thiazolidinediones, dipeptidyl peptidase-4 inhibitors, α-glucosidase inhibitors, and insulin). |
| Dyslipidemia | E78; and claims for agents for dyslipidemia or Total cholesterol ≥ 240 |
| Chronic obstructive pulmonary disease | J41-J44 |
| End-stage renal disease | N18, N19, Z49, Z940, Z90, Z992; and obligatory accompanied with either procedure claims or benefit extension policy claims for hemodialysis, peritoneal dialysis or renal transplantation |
| Peripheral artery disease | I70, I73 |
| **Cardiovascular events** |  |
| Myocardial infarction | I21,22; and hospitalization ≥ 1 |
| Atrial fibrillation | I48.0-I48.4, I48.9; and Hospitalization ≥ 1 or Outpatient visit ≥ 2 with excluding mitral stenosis (I05.0, I05.2, I05.9) and mechanical heart valves (Z95.2-Z95.4) |
| Heart Failure | I50; and hospitalization ≥ 1 |
| Ischemic stroke | I63, I64; Hospitalization ≥ 1 with claims for the imaging studies (brain CT or MRI) |

^a^ Hypertension and diabetes mellitus were identified when patients had ≥1 diagnoses during hospitalization or ≥2 diagnoses at an outpatient clinic for preventing overestimation of diagnosis.
